# Declines in Influenza Vaccination Coverage Among Health Care Personnel in Acute Care Hospitals During the COVID-19 Pandemic — United States, 2017–2023

**DOI:** 10.15585/mmwr.mm7245a6

**Published:** 2023-11-10

**Authors:** Hoody Lymon, Lu Meng, Hannah E. Reses, Kira Barbre, Heather Dubendris, Shanjeeda Shafi, Ryan Wiegand, Gnanendra Reddy Tugu Yagama Reddy, Austin Woods, David T. Kuhar, Matthew J. Stuckey, Megan C. Lindley, Lori Haas, Iram Qureshi, Emily Wong, Andrea Benin, Jeneita M. Bell

**Affiliations:** ^1^Division of Healthcare Quality Promotion, National Center for Emerging and Zoonotic Infectious Diseases, CDC; ^2^Oak Ridge Institute for Science and Education, Oak Ridge, Tennessee; ^3^Goldbelt C6, Chesapeake, Virginia; ^4^Lantana Consulting Group, East Thetford, Vermont; ^5^Coronavirus and Other Respiratory Viruses Division, National Center for Immunization and Respiratory Diseases, CDC; ^6^Chenega Enterprise Systems & Solutions, LLC, Chesapeake, Virginia; ^7^Immunization Services Division, National Center for Immunization and Respiratory Diseases, CDC; ^8^Leidos, Inc., Atlanta, Georgia.

**Summary**

**What is already known about this topic?**

CDC and the Advisory Committee on Immunization Practices recommend that health care personnel (HCP) receive an annual influenza vaccination to reduce influenza-related morbidity and mortality.

**What is added by this report?**

HCP working in acute care hospitals during the 2017–2023 influenza seasons were less likely to be vaccinated against influenza during the COVID-19 pandemic (influenza seasons 2020–21 through 2022–23) than before the pandemic (influenza seasons 2017–18 through 2019–20).

**What are the implications for public health practice?**

Efforts are needed to implement evidence-based strategies to increase vaccination coverage among HCP. HCP should receive seasonal influenza vaccines to protect other HCP and patients from influenza-related morbidity and mortality.

## Abstract

Health care personnel (HCP) are recommended to receive annual vaccination against influenza to reduce influenza-related morbidity and mortality. Every year, acute care hospitals report receipt of influenza vaccination among HCP to CDC’s National Healthcare Safety Network (NHSN). This analysis used NHSN data to describe changes in influenza vaccination coverage among HCP in acute care hospitals before and during the COVID-19 pandemic. Influenza vaccination among HCP increased during the prepandemic period from 88.6% during 2017–18 to 90.7% during 2019–20. During the COVID-19 pandemic, the percentage of HCP vaccinated against influenza decreased to 85.9% in 2020–21 and 81.1% in 2022–23. Additional efforts are needed to implement evidence-based strategies to increase vaccination coverage among HCP and to identify factors associated with recent declines in influenza vaccination coverage.

## Introduction

Health care personnel (HCP), including those working in acute care hospitals, are at risk for becoming infected with influenza, missing work due to illness, and transmitting the virus to their patients and to other staff members (*1*). The Advisory Committee on Immunization Practices recommends that HCP receive an annual influenza vaccine to reduce influenza-associated morbidity and mortality (*2*). In 2013, the Centers for Medicare & Medicaid Services (CMS) began requiring acute care hospitals to report aggregate facility-level data on HCP influenza vaccination to CDC’s National Healthcare Safety Network (NHSN) (*3*). This analysis used data reported to NHSN to assess annual variation in influenza vaccination coverage among HCP during six influenza seasons (2017–18 through 2022–23) to describe annual changes in influenza vaccination coverage among HCP before and during the COVID-19 pandemic.

## Methods

### Data Collection

Since 2013, every year, acute care hospitals report aggregate facility-level influenza vaccination data among HCP working in the facility for ≥1 day during October 1–March 31 to NHSN.* During the 2019–20 influenza season, CMS suspended the NHSN reporting requirement to reduce regulatory workload during the COVID-19 pandemic,^†^ making reporting optional during this season. This report includes data submitted by acute care hospitals to NHSN for the six influenza seasons from 2017–18 to 2022–23.

### Data Analysis

For each influenza season, both pooled HCP influenza vaccination coverage (calculated by dividing the number of HCP who received the annual influenza vaccine by the total number of HCP among all reporting facilities) and facility-level coverage (calculated by dividing the reported number of HCP who worked at a specific facility and received the annual influenza vaccine by the total number of HCP who worked at the facility) were examined. A logistic regression model with generalized estimating equations was developed to assess differences in influenza vaccination coverage before the COVID-19 pandemic (prepandemic) and during the pandemic, controlling for HCP type (employee [those receiving a paycheck directly from the health care facility], nonemployee licensed practitioner [physicians, advanced practice nurses, and physician assistants who are affiliated with the health care facility, but are not directly employed by it], and nonemployee students and volunteer [medical, nursing, or other health professional students, interns, medical residents, or volunteers aged ≥18 years who are affiliated with the health care facility but are not directly employed by it]),^§^ three levels of urbanicity of the hospital location (urban, suburban, and rural),^¶^ and the six influenza seasons (2017–18 through 2022–23).

The prepandemic period included influenza seasons 2017–18 through 2019–20, and the pandemic period included seasons 2020–21 through 2022–23, with the 2017–18 season set as the reference season. The 2019–20 season was included with the prepandemic seasons because influenza vaccination is generally recommended by the end of October,** and most influenza vaccines are administered by January.^††^ Thus, because widespread community transmission of SARS-CoV-2 in the United States did not begin until March 2020 (*4*), the pandemic is unlikely to have affected influenza vaccination during that season. In considering job category, the reference group was set as employee; for urbanicity, the reference group was rural. Descriptive analyses were performed at the facility level, with median coverage levels and IQRs calculated by season. Pooled facility-level vaccination coverage was further stratified by each factor included in the generalized estimating equations model, and odds ratios (OR) and 95% CI were calculated. P-values <0.05 and 95% CI that excluded 1 were considered statistically significant. All analysis was conducted using SAS (version 9.4; SAS Institute). This activity was reviewed by the CDC, deemed not research, and conducted consistent with applicable federal law and CDC policy.^§§^

## Results

During the 2017–18 through 2022–23 influenza seasons, 5,231 acute care hospitals reported HCP influenza vaccination data to NHSN (Table 1), including 2,908 (55.6%) hospitals during the 2019–20 season, when reporting was optional. During the six-season period, overall pooled influenza vaccination coverage among HCP was 85.8%. During the prepandemic influenza seasons (2017–18, 2018–19, and 2019–20), pooled annual influenza vaccination coverage was 88.6%, 90%, and 90.7%, respectively. After the emergence of SARS-CoV-2, coverage during the 2020–21, 2021–22, and 2022–23 seasons declined to 85.9%, 80.4%, and 81.1%, respectively.

**TABLE 1:** Pooled and facility-level influenza vaccination coverage among health care personnel in acute care hospitals, by influenza season, employee type, and urbanicity* — National Healthcare Safety Network, United States, 2017–18 through 2022–23 influenza seasons

Season/HCP type/Urbanicity	Pooled coverage				Facility-level coverage, %, median (IQR)
	No. of facilities	No. of HCP	No. of HCP vaccinated	Vaccination coverage, %	
**Total**	**5,231**	**48,082,901**	**41,246,748**	**85.8**	**93.21 (76.00–98.87)**
**Influenza season**					
**Prepandemic period**					
2017–18	4,735	8,661,994	7,678,088	88.6	94.12 (80.64–98.89)
2018–19	4,655	8,715,667	7,840,603	90.0	95.20 (84.16–99.10)
2019–20	2,908	5,167,211	4,684,995	90.7	95.47 (85.23–99.26)
**Pandemic period**					
2020–21	4,469	8,217,159	7,059,341	85.9	93.86 (77.76–99.38)
2021–22	4,602	8,530,075	6,856,292	80.4	89.48 (64.44–98.27)
2022–23	4,759	8,790,795	7,127,429	81.1	88.23 (65.12–97.48)
**HCP type**					
Employee	5,231	35,633,277	31,364,746	88.0	92.06 (80.49–97.07)
Licensed independent practitioner	5,231	6,935,148	5,033,055	72.6	87.75 (57.98–98.25)
Student trainee or volunteer	5,231	5,514,476	4,848,947	87.9	98.22 (86.36–100.00)
Urbanicity*					
Rural	1,883	5,342,766	4,597,676	86.1	93.75 (76.33–99.83)
Suburban	1,439	16,285,637	14,141,350	86.8	93.45 (77.08–98.73)
Urban	1,869	26,294,322	22,369,551	85.1	92.61 (75.00–98.44)

**Abbreviation**: HCP = health care personnel.

* Urbanicity categories are based on the National Center for Health Statistics’ rural-urban classification scheme for counties. Urban areas include counties within metropolitan statistical areas of ≥1 million population, suburban areas include counties within metropolitan statistical areas of 50,000–999,999 population, and rural areas include counties outside of metropolitan statistical areas (including counties within and outside of micropolitan statistical areas). https://www.cdc.gov/nchs/data/data_acces_files/NCHSUrbruralFileDocumentationInternet2.pdf

When controlled for HCP type and urbanicity, HCP were significantly less likely to be vaccinated against influenza during the pandemic than they were during the 2017–18 season, (Table 2). Across all influenza seasons, compared with HCP employed by hospitals, influenza vaccination coverage was lower among licensed independent practitioners (OR = 0.35; 95% CI = 0.34–0.37) and student trainees and volunteers (OR = 0.98; 95% CI = 0.81–0.98). Compared with HCP working in rural areas, HCP working in suburban areas were more likely to be vaccinated (OR = 1.16; 95% CI = 1.05–1.28).

**TABLE 2:** Influenza vaccination coverage differences among health care personnel in acute care hospitals,* by influenza season, health care personnel type, and urbanicity^†^ — National Healthcare Safety Network, United States, 2017–18 through 2022–23 influenza seasons

Influenza season	Odds ratio (95% CI)	p-value
**Prepandemic period**		
2017–18	Ref	—
2018–19	1.14 (1.10–1.19)	<0.01
2019–20	1.24 (1.17–1.31)	<0.01
**Pandemic period**		
2020–21	0.76 (0.72–0.80)	<0.01
2021–22	0.50 (0.47–0.53)	<0.01
2022–23	0.52 (0.49–0.55)	<0.01
**HCP type**		
Employee	Ref	—
Licensed independent practitioner	0.35 (0.34–0.37)	<0.01
Student trainee or volunteer	0.89 (0.81–0.98)	0.02
**Urbanicity^†^**		
Rural	Ref	—
Suburban	1.16 (1.05–1.28)	<0.01
Urban	1.03 (0.94–1.14)	0.45

**Abbreviations**: HCP = health care personnel; Ref = referent group.

* Based on a generalized estimating equations model, controlling for health care provider type and urbanicity.

^†^ Urbanicity categories are based on the National Center for Health Statistics’ rural-urban classification scheme for counties. Urban areas include counties within metropolitan statistical areas of ≥1 million population, suburban areas include counties within metropolitan statistical areas of 50,000–999,999 population, and rural areas include counties outside of metropolitan statistical areas (including counties within and outside of micropolitan statistical areas). https://www.cdc.gov/nchs/data/data_acces_files/NCHSUrbruralFileDocumentationInternet2.pdf

## Discussion

This study of a large national surveillance system found that influenza vaccination coverage among HCP in acute care hospitals has declined since the COVID-19 pandemic. A recent study reported similar results from an Internet panel survey of HCP in which estimated influenza vaccination coverage based on self-reported receipt of influenza vaccine decreased between the 2019–20 and the 2020–21 seasons (*5*). These findings underscore the importance of investigating reasons for declines in vaccination coverage among HCP.

A combination of factors might have affected the decline in influenza vaccination coverage among HCP during the COVID-19 pandemic. Similar decreases in influenza vaccination observed among HCP in other countries have been attributed to COVID-19 vaccination campaigns leading to decreased emphasis on influenza vaccination and vaccine fatigue from having received multiple COVID-19 vaccines (*6*). Although the co-administration of COVID-19 and influenza vaccines is safe and effective,^¶¶^ individual hesitancy to receive both vaccines at once might also have contributed to lower influenza vaccination coverage during the pandemic. In the United States, facility-wide mask-wearing was recommended by CDC during the pandemic,*** and fewer influenza cases, hospitalizations, and deaths were reported during the 2020–21 and 2021–22 influenza seasons compared with previous years.^†††^ These factors might have contributed to perceptions that the risk for acquiring influenza in the workplace was lower and that influenza vaccination was less important than it had been in previous years.

Similar to reports from previous influenza seasons, nonemployee licensed independent practitioners were consistently found to have lower vaccination coverage compared with employees.^§§§^ Personnel directly employed by a hospital might be more easily reached by strategies to increase vaccination, such as offering vaccination at no cost, eliminating an important barrier to access. In addition, facility-issued vaccine mandates have been found to improve HCP influenza vaccination coverage (*7*). However, facility-level vaccination mandates might not be easily enforced among nonemployee licensed independent practitioners, potentially limiting the effectiveness of this strategy in this subpopulation of HCP.

Previous reports of HCP vaccination have found that HCP working in rural areas had lower vaccination coverage compared with those working in nonrural areas (*8*). This report found that, compared with influenza vaccination coverage among HCP working in rural areas, influenza vaccination coverage among those working in suburban areas was significantly higher. There was no difference in coverage among HCP working in urban and rural areas. This suggests that future studies of vaccination coverage might consider examining HCP working in suburban and urban areas as separate subgroups.

### Limitations

The findings in this report are subject to at least four limitations. First, this report includes influenza vaccination data reported by facilities to NHSN on behalf of HCP, which could have resulted in an underestimate of influenza vaccination acquired by HCP outside the hospital, particularly among HCP not employed directly by the reporting hospital. Second, NHSN received aggregate facility-level data; therefore, vaccination coverage could not be stratified by person-level covariates including age, race, or ethnicity. Third, a reporting exception was granted for acute care hospitals for the 2019–2020 season, resulting in only a subset of facilities reporting and limiting the generalizability of the data reported that year. Finally, NHSN does not collect data about facility-level influenza vaccination mandates for HCP. Therefore, it was not possible to determine the impact that facility-level vaccination mandates or changes to mandates over time might have had on influenza vaccination coverage among HCP.

### Implications for Public Health Practice

Receipt of an annual influenza vaccination by HCP working in acute care hospitals is important for protecting themselves and hospitalized patients from influenza infection and its associated complications. Acute care hospitals can use evidence-based strategies to increase vaccination coverage, including implementing mandatory immunization policies and offering on-site influenza vaccination at no cost to all employee and nonemployee staff members.^¶¶¶^ Understanding factors contributing to recent declines in influenza vaccination among HCP might facilitate targeted interventions to increase influenza vaccination coverage during future public health emergencies.

## Footnotes

*
https://www.cdc.gov/nhsn/pdfs/hps-manual/vaccination/hps-flu-vaccine-protocol-508.pdf

†
https://www.cms.gov/files/document/guidance-memo-exceptions-and-extensions-quality-reporting-and-value-based-purchasing-programs.pdf

§
https://www.cdc.gov/nhsn/forms/instr/COVIDVax.Staff_.Revised.TOI_.MAY2022-508.pdf

¶ Urbanicity categories are based on the National Center for Health Statistics’ rural-urban classification scheme for counties. Urban areas include counties within metropolitan statistical areas of ≥1 million population, suburban areas include counties within metropolitan statistical areas of 50,000–999,999 population, and rural areas include counties outside of metropolitan statistical areas (including counties within and outside of micropolitan statistical areas). https://www.cdc.gov/nchs/data/data_acces_files/NCHSUrbruralFileDocumentationInternet2.pdf

**
https://www.cdc.gov/flu/professionals/vaccination/vax-summary.htm

††
https://www.cdc.gov/flu/fluvaxview/interactive-general-population.htm

§§ 45 C.F.R. part 46.102(l)(2), 21 C.F.R. part 56; 42 U.S.C. Sect. 241(d); 5 U.S.C. Sect. 552a; 44 U.S.C. Sect. 3501 et seq.

¶¶
https://www.cdc.gov/flu/prevent/coadministration.htm

***
https://www.cdc.gov/flu/about/burden/past-seasons.html

†††
https://www.cdc.gov/coronavirus/2019-ncov/hcp/infection-control-recommendations.html

§§§
https://www.cdc.gov/flu/fluvaxview/interactive-health-care-personnel.htm

¶¶¶
https://www.cdc.gov/infectioncontrol/guidelines/healthcare-personnel/immunization.html
